# 

**Published:** 1903-10

**Authors:** 


### BOOKS RECEIVED.

Nurses' Guide to Surgical Bandging and Dressings. By William Johnson Smith, F. R. C. S., Principal Medical Officer, Seamen's Hospital, Greenwich. Philadelphia: J. B. Lippincott Co. 1903. (Price, 75 cents.)

Diseases of the Nose and Throat. By Charles Huntoon Knight, A. M., M. D., Professor of Laryngology, Cornell University Medical College. Octavo, 438 pages; 147 illustrations. Philadelphia: P. Blakiston's Son & Co. 1903. (Price, $3.00.)

Transactions of the Medical Society of the State of New York. Ninety-seventh Annual Meeting, held at Albany, N. Y., January 27, 28 and 29, 1903. Frederic C. Curtis, M. D., Secretary. Brandon Printing Co., Albany. 1903.

Progressive Medicine. Fifth Annual Series. Volume III. September, 1903. A Quarterly Digest of Advances, Discoveries and Improvements in the Medical and Surgical Sciences. Edited by Hobart Amory Hare, M. D., Professor of Therapeutics and Materia Medica in the Jefferson Medical College of Philadelphia. Octavo, 398 pages; illustrated. Philadelphia and New York: Lea Brothers & Co. (Per volume, $2.50, by express prepaid. Per annum, in four cloth-bound volumes, $10.00.)

A System of Physiologic Therapeutics. A Practical Exposition of the Methods, other than Drug-giving, useful for the Prevention of Disease and in the Treatment of the Sick. Edited by Solomon Solis Cohen, A. M., M. D. Senior Assistant Professor of Clinical Medicine in Jefferson Medical College. Volume VIII. Rest, Mental Therapeutics, Suggestion. By Francis X. Dercum, M. D., Professor of Nervous and Mental Diseases in the Jefferson Medical College of Philadelphia. Octavo, 332 pages. Philadelphia: P. Blakiston's Son & Co. 1903. (Price for the set, $27.50 net.)

Scheme for the Differential Testing of Nerves and Muscles. For use in Diagnosis. By J. Montgomery Mosher, M. D., Clinical Professor of Insanity, Neurology and Electrotherapeutics, Albany Medical College. Octavo, 58 pages; illustrated. Albany: Brandon Printing Co. 1903. (Price, $1.00.)

Blakiston's Quiz Compends. A Compend of Human Anatomy. By Samuel O. L. Potter, M. D., Author of the Handbook of Materia Medica, Pharmacy and Therapeutics. Seventh edition, revised and enlarged. Illustrated. Philadelphia: P. Blakiston's Son & Co. 1903. (Price, 80 cents.)

Blakiston's Quiz Compends. A Compend of Diseases of the Skin. By Jay F. Schamberg, A. B., M. D., Professor of Diseases of the Skin, Philadelphia Polyclinic and College for Graduates in Medicine. Third edition, revised and enlarged, with 106 illustrations. Philadelphia: P. Blakiston's Son & Co. 1903. (Price, 80 cents.)

Manual of the Diseases of the Eye. For Students and General Practitioners. By Charles H. May, M. D., Chief of Clinic and Instructor in Ophthalmology, College of Physicians and Surgeons, Medical Department, Columbia University, New York. Third edition, revised. Duodecimo, 421 pages, with 275 original illustrations, including 16 plates, with 36 colored figures. New York: William Wood & Co. 1903. (Price, $2.00.)

